# Single‐Shot Intrathecal Bupivacaine With Fentanyl vs. Dexmedetomidine for Labor Analgesia: A Randomized Controlled Trial

**DOI:** 10.1155/anrp/6685169

**Published:** 2026-04-29

**Authors:** Ayman Mohamady Eldemrdash, Taha Tairy Dardeer Elsawy, Zaher Zaki Zaher, Jehan Sadek Younan, Ahmed Mohammed Reda Ragheb

**Affiliations:** ^1^ Anesthesiology, Surgical Intensive Care and Pain Medicine Department, Faculty of Medicine, Aswan University, Aswan, Egypt, aswu.edu.eg

**Keywords:** dexmedetomidine, fentanyl, intrathecal bupivacaine, labor analgesia, maternal satisfaction, single-shot spinal anesthesia, visual analog scale (VAS)

## Abstract

**Background:**

Labor pain is one of the most intense physiological pains experienced by women. Single‐shot spinal analgesia (SSSA) offers rapid and reliable labor pain relief. Combining bupivacaine with adjuvants like fentanyl or dexmedetomidine has been proposed to enhance analgesic efficacy and maternal comfort.

**Methods:**

In this randomized, double‐blind clinical trial, 90 full‐term parturients in active labor were randomly allocated into three equal groups: Group B: 2.5 mg intrathecal bupivacaine with 0.5 mL of saline; Group BF: 2.5 mg bupivacaine with 25 μg fentanyl; Group BD: 2.5 mg bupivacaine with 5 μg dexmedetomidine. We compared the duration and onset of analgesia, pain scores on the visual analog scale (VAS), maternal satisfaction using the short assessment of patient satisfaction (SAPS), adverse effects, and neonatal outcomes.

**Results:**

Group BD demonstrated the fastest onset of analgesia (2.1 ± 0.7 min) and the longest duration (124.2 ± 15.4 min), compared to Group BF (3.6 ± 0.9 min; 106.2 ± 18.9 min) and Group B (4.2 ± 1.0 min; 80.4 ± 14.6 min) (*p* < 0.001). The pattern of pain reduction over time varied across treatments; Group BD showed the most rapid and sustained decrease in pain intensity, while Group B exhibited the slowest decline. A significant time × group interaction (*p* < 0.001, partial *η* ^2^ = 0.67, i.e., large effect size) was observed. Maternal satisfaction scores were significantly higher in Group BD (26.63 ± 2.03) than in BF (22.00 ± 3.29) or B (15.27 ± 3.68) (*p* < 0.01). The incidence of itching and nausea was higher in the BF group, and mild bradycardia was found in the BD group. Apgar scores at 1 and 5 min were similar in all groups.

**Conclusion:**

Dexmedetomidine added to intrathecal bupivacaine produced good labor analgesia with high maternal satisfaction, with low side effects, and could be used as a better substitute for fentanyl.

**Trial Registration:** ClinicalTrials.gov: NCT06823349

## 1. Introduction

Because of the intricate interplay between visceral and somatic pain stimuli that takes place during uterine contractions and the fetus’s descent through the birth canal, labor is frequently referred to as one of the most agonizing physiological events in a woman’s life [1]. Significant maternal stress reactions, such as hyperventilation, catecholamine release, and impaired uteroplacental perfusion, can result from this severe pain, and these reactions may have detrimental effects on the mother and fetus [2]. Even in the absence of medical indications, women in many low‐ and middle‐income countries choose elective cesarean deliveries due in large part to their fear of labor pains [3].

Therefore, it is essential to manage labor pain effectively to promote a safe vaginal delivery as well as the comfort of the mother. The best ways to relieve labor pain are generally acknowledged to be neuraxial techniques, particularly spinal and epidural analgesia [4]. Among these, single‐shot spinal analgesia (SSSA) has become more well‐liked because of its affordability, quick onset, high success rate, and ease of administration [5, 6]. However, spinal analgesia’s main drawback is its comparatively brief duration of action.

Because of its dependable sensory blockade, low placental transfer, and favorable safety profile, bupivacaine, a long‐acting amide local anesthetic, is frequently administered intrathecally [7]. Higher dosages, however, may result in unintended motor blockade, which could impair the mother’s mobility and ability to participate in the second stage of labor actively. Adjuvants are frequently used in conjunction with bupivacaine to improve the quality of analgesia while reducing motor block. For this, fentanyl, a strong *μ*‐opioid receptor agonist, is frequently utilized. By interacting with opioid receptors in the spinal cord, it reduces the amount of local anesthetic needed to provide quick and efficient pain relief [8]. However, there is still worry about opioid‐related adverse effects like respiratory depression, nausea, and pruritus. A promising substitute adjuvant is dexmedetomidine, a selective *α*2adrenergic receptor agonist. By preventing the release of substance P and norepinephrine, it reduces nociceptive transmission and produces analgesic effects via both spinal and supraspinal mechanisms [9]. Unlike opioids, dexmedetomidine provides sedation and analgesia without causing respiratory depression, making it an attractive option for labor analgesia [10].

Given these pharmacologic profiles, this study was designed to evaluate and compare the effectiveness of intrathecal bupivacaine alone, bupivacaine with fentanyl, and bupivacaine with dexmedetomidine in terms of analgesic efficacy, onset and duration of action, maternal satisfaction, side effects, and neonatal outcomes during normal labor.

## 2. Methods

### 2.1. Study Design

This study was a prospective, randomized, double‐blind, controlled clinical trial conducted at the labor unit of Aswan University Hospital between February 15, 2025, and July 1, 2025. The study protocol was approved by the institutional ethics committee (Approval No: Asw\.Uni./926/5/24). All participants provided informed written consent before enrollment, in accordance with the Declaration of Helsinki.

### 2.2. Participants

A total of 90 full‐term parturients aged 18–40 years, with American Society of Anesthesiologists (ASA) Physical Status II and a singleton fetus in cephalic presentation, were recruited. All were in active labor with cervical dilatation > 5 cm and requested neuraxial analgesia.

Inclusion Criteria: ASA II, term pregnancy with singleton fetus, cervical dilation > 5 cm, and cephalic fetal presentation.

Exclusion Criteria: Patient refusal, known allergy to study drugs, bleeding disorders or use of anticoagulants, infection at the injection site, and spinal deformities.

These criteria were selected based on previous labor analgesia research to ensure safety and uniformity among study groups [4, 5].

### 2.3. Randomization and Blinding

Participants were randomly assigned to three equal groups (*n* = 30 each) using a computer‐generated randomization sequence with 1:1:1 allocation. Allocation concealment was maintained using opaque sealed envelopes. Both participants and outcome assessors were blinded to the group assignment to minimize bias [11].

### 2.4. Study Drug Preparation

To ensure identical total intrathecal volume and maintain blinding, all study solutions were prepared by an independent anesthesia technician in a standardized manner. Plain 0.5 mL bupivacaine (2.5 mg) was drawn and combined with either 0.5 mL of preservative‐free normal saline (Group B), 0.5 mL containing 25 μg fentanyl (Group BF), or 0.5 mL containing 5 μg dexmedetomidine (Group BD), giving a total intrathecal volume of 1.0 mL for every participant. For fentanyl and dexmedetomidine, stock solutions were diluted with preservative‐free saline to the specified 0.5 mL volumes using sterile technique and single‐use syringes; drug concentrations and final volumes were double‐checked by two staff members before administration. This procedure maintained equal volume across groups and preserved blinding.

### 2.5. Study Groups

Group B (Control): 2.5 mg (0.5 mL) bupivacaine + 0.5 mL saline.

Group BF (Fentanyl): 2.5 mg (0.5 mL) bupivacaine + 25 μg (0.5 mL) fentanyl.

Group BD (Dexmedetomidine): 2.5 mg (0.5 mL) bupivacaine + 5 μg (0.5 mL) dexmedetomidine.

Stock solutions of fentanyl and dexmedetomidine were diluted using sterile technique with single‐use syringes. Two staff members independently verified drug concentrations and final volumes before administration. Identical syringes were used across all groups, and the administering anesthesiologist was blinded to group allocation throughout the study.

### 2.6. Intervention

The SSSA technique was performed under strict aseptic precautions. With the patient in a sitting position, a 25‐gauge Quincke spinal needle was inserted at the L3–L4 interspace. After confirming free flow of cerebrospinal fluid, 1 mL of the allocated study solution was administered intrathecally. Patients were then positioned supine with a wedge under the right hip to prevent aortocaval compression.

Standard monitoring included noninvasive blood pressure (NIBP), heart rate (HR), respiratory rate (RR), and oxygen saturation (SpO_2_), recorded at 5‐min intervals for the first 30 min, then every 20 min for the next 3 h, followed by hourly measurements. Pain was assessed using a 10‐point visual analog scale (VAS) (0 = no pain, 10 = worst pain imaginable) at baseline, then every 5 min for the first 30 min, every 20 min for 3 h, and every 30 min thereafter.

Hypotension (defined as > 25% drop in MAP) was treated with 5 mg IV ephedrine, and bradycardia (HR < 50 bpm) was managed with 0.5 mg atropine. If VAS ≥ 8, 50 mg of intramuscular pethidine was administered.

Continuous fetal HR monitoring was performed via cardiotocography (CTG) from the time of intrathecal injection until delivery, as per standard labor unit protocol.

### 2.7. Outcome Measures

#### 2.7.1. Primary Outcomes

Onset of analgesia (time from injection to VAS ≤ 4), duration of analgesia (time from onset to need for rescue analgesia), and pain intensity measured via VAS over time.

#### 2.7.2. Secondary Outcomes

Maternal satisfaction assessed using the short assessment of patient satisfaction (SAPS) is a validated questionnaire designed to measure patient satisfaction with healthcare services, scored on a scale from 11 to 28, and was done postdelivery (11–18 = dissatisfied, 19–26 = satisfied, 27–28 = very satisfied) [12].

Maternal hemodynamic parameters: MAP, HR, SpO_2_, RR.

Adverse effects: nausea, vomiting, pruritus, bradycardia, hypotension.

Neonatal outcome: assessed by Apgar scores at 1 and 5 min.

### 2.8. Sample Size

Sample size is calculated by the GPower software Version 3.1.3 program based on data from a previous study, according to a previously published study (Jain et al., Anesthesia, essays and research, volume 16, Apr‐Jun 2022) [7]. With the two‐sided *α* error set at 0.05 and the *β* error set at 0.2 (power of 80%), the mean and standard deviation (SD) of the primary outcome were compared to determine the duration and onset of analgesia in vaginal delivery. The mean and SD in the fentanyl group were mean (106.24) minutes and SD (18.9), and in the dexmedetomidine group were mean (124.16) minutes and SD (26.23). The sample size is 27 patients per group, and based on this value, the sample size is taken for the primary outcome, 81 cases (27 patients in each group). Taking into account a 10% loss to follow‐up, we decided to recruit 30 patients per group. To account for potential dropouts and challenging cases, an additional 3 patients were added to each group, resulting in a total sample size of 90 patients (30 per group).

### 2.9. Statistical Analysis

Data analysis was performed using IBM SPSS Statistics Version 26. Quantitative variables were expressed as mean ± SD, and qualitative data as frequencies and percentages. Between‐group comparisons were done using one‐way ANOVA for continuous variables, followed by Bonferroni post hoc tests for pairwise comparisons. Categorical variables were analyzed using the chi‐square test or Fisher’s exact test as appropriate. Repeated measures of VAS scores and hemodynamic variables were analyzed using repeated‐measures ANOVA (RM‐ANOVA), with the Greenhouse–Geisser correction applied when the assumption of sphericity was violated. A *p* value < 0.05 was considered statistically significant.

## 3. Results

**FIGURE 1:**
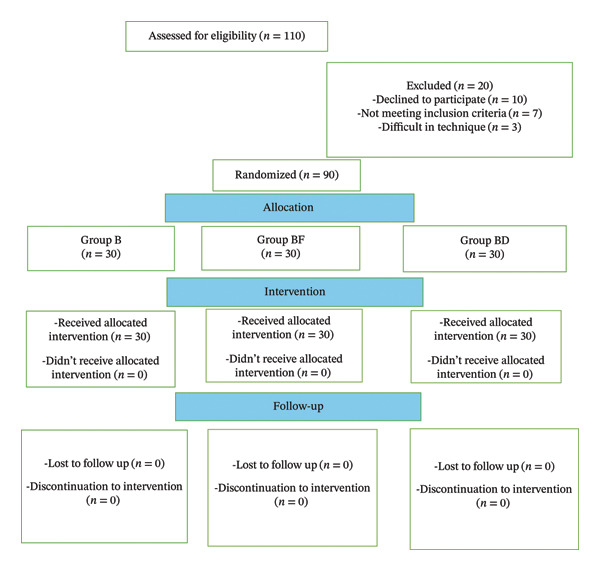
CONSORT flowchart.

Out of 110 laboring parturient assessed for eligibility, 90 women met the inclusion criteria and were randomized into three equal groups of 30 each. Twenty participants were excluded: 10 declined participation, seven did not meet the eligibility criteria, and three had difficulty technically with the spinal anesthesia. All 90 allocated participants completed the study protocol and were included in the final analysis, with no loss to follow‐up or protocol deviations (Figure 1).

The baseline demographic and obstetric characteristics were comparable among the three groups. There were no statistically significant differences regarding age, weight, height, body mass index (BMI), gestational age, or parity (*p* > 0.05). This confirmed successful randomization and group equivalence at baseline (Table 1).

**TABLE 1:** Demographic, obstetric, and baseline parameters across study groups.

Parameters	Group B control (*n* = 30)	Group BF fentanyl (*n* = 30)	Group BD dexmedetomidine (*n* = 30)	*p* value
	Mean ± SD or *n* (%)			
Age (years)	24.47 ± 3.25	24.97 ± 3.59	25.10 ± 3.93	0.773
Weight (kg)	86.03 ± 7.26	86.77 ± 7.05	84.03 ± 8.73	0.369
Height (cm)	159.77 ± 3.55	160.57 ± 3.34	160.07 ± 3.27	0.654
BMI (kg/m^2^)	33.67 ± 2.10	33.66 ± 2.58	32.79 ± 3.19	0.350
Gestational age (weeks)	38.93 ± 0.87	38.83 ± 0.91	38.57 ± 1.07	0.312
Parity				
Primigravida	25 (83.3)	24 (80.0)	25 (83.3)	0.932
Multigravida	5 (16.7)	6 (20.0)	5 (16.7)	
ASA II	30 (100.0)	30 (100.0)	30 (100.0)	—

*Note:* Values are mean ± SD or numbers (percentage). All statistical tests were two‐tailed. Test used = one‐way ANOVA and chi‐square; *p* < 0.05 is significant.

Abbreviation: SD, standard deviation.

Regarding analgesic efficacy, the time to achieve effective analgesia (defined as time to VAS ≤ 4) was significantly shorter in the group that received bupivacaine with dexmedetomidine (Group BD), with a mean onset of 2.1 ± 0.7 min. This was significantly faster than both the fentanyl group (Group BF) at 3.6 ± 0.9 min and the control group receiving bupivacaine alone (Group B) at 4.2 ± 1.0 min (*p* < 0.001). Similarly, the duration of analgesia was significantly prolonged in Group BD, with a mean of 124.2 ± 15.4 min, followed by Group BF at 106.2 ± 18.9 min, and the shortest duration was observed in Group B at 80.4 ± 14.6 min (*p* < 0.001). These findings highlight the superior duration and rapid onset of analgesia provided by dexmedetomidine as an adjuvant (Table 2). Labor duration was nonsignificant regarding the three groups (*p* 0.21), since analgesia type does not meaningfully alter labor progression. No significant correlation was observed between duration of spinal analgesia and time to delivery, Spearman *ρ* = 0.11 (*p* = 0.29).

**TABLE 2:** Comparison of maternal outcomes among study groups using one‐way ANOVA and Bonferroni post hoc tests.

Parameter	Group B control (*n* = 30)	Group BF fentanyl (*n* = 30)	Group BD dexmedetomidine (*n* = 30)	*p* value	Bonferroni post hoc pairwise comparison	Mean difference (95% CI)^a^
	(Mean ± SD)					
Onset to VAS ≤ 4 (min.)	4.2 ± 1.0	3.6 ± 0.9	2.1 ± 0.7	**< 0.001**	*P*1 < 0.001	# 2.15 (1.82–2.48)
					*P*2 < 0.001	## 2.03 (1.68–2.38)
					*P*3 = 0.511 (NS)	### − 0.12 (−0.47–0.23)
Duration of analgesia (min.)	80.4 ± 14.6	106.2 ± 18.9	124.2 ± 15.4	**< 0.001**	*P*1 < 0.001	# − 46.37 (−53.26 to −39.48)
					*P*2 < 0.001	## − 20.07 (−27.71–−12.43)
					*P*3 < 0.001	### 26.30 (17.10–35.50)
Labor duration (time from injection to delivery/min)	82 ± 18	78 ± 20	86 ± 22	0.21 (NS)	—	
Time to S1 regression (min.)	40.67 ± 6.26	50.33 ± 8.60	59.67 ± 8.90	**< 0.001**	*P*1 < 0.001	# − 19.00 (−22.98 to −15.02)
					*P*2 < 0.001	## − 9.66 (−13.55–−5.77)
					*P*3 < 0.001	### 9.34 (4.82–13.86)
Maternal Satisfaction Score	15.27 ± 3.68	22.00 ± 3.29	26.63 ± 2.03	**0.003**	*P*1 < 0.001	# – 3.36 (– 4.90–−1.82)
					*P*2 = 0.163 (NS)	## − 1.73 (– 3.53–0.07)
					*P*3 = 0.071 (NS)	### 1.63 (0.22–3.04)

*Note:* Data are presented as mean ± SD; (*P*1 & #): between Group B and Group BF, (*P*2 & ##): between Group B and Group BD, (*P*3 & ###): between Group BF and Group BD. Bold represents significant *p* value.

Abbreviation: CI, confidence interval.

^a^Using one‐way ANOVA and Bonferroni post hoc test comparisons, one‐way ANOVA test and two‐tailed *p* value *t*‐statistic were used.

Time to regression of the sensory block to the S1 dermatome was significantly prolonged in the group receiving intrathecal bupivacaine with dexmedetomidine (Group BD), with a mean of 59.67 ± 8.90 min, compared to 50.33 ± 8.60 min in the fentanyl group (Group BF) and 40.67 ± 6.26 min in the control group (Group B) (*p* < 0.001). Pairwise comparisons revealed statistically significant differences among all groups: Group BD vs. B (*p* < 0.001), BD vs. BF (*p* < 0.001), and BF vs. B (*p* < 0.001). These findings indicate that dexmedetomidine not only prolonged analgesic duration but also delayed sensory regression more effectively than fentanyl or bupivacaine alone (Table 2).

Maternal satisfaction, as measured by the SAPS questionnaire, was highest in Group BD, where the majority of participants reported being “very satisfied,” with a mean score of 26.63 ± 2.03. Group BF showed moderate satisfaction (22.00 ± 3.29), while Group B participants were mostly “dissatisfied,” with a mean SAPS score of 15.27 ± 3.68. These differences were statistically significant (*p* = 0.003), reinforcing the clinical impact of effective analgesia on patient‐reported outcomes (Table 2).

**FIGURE 2:**
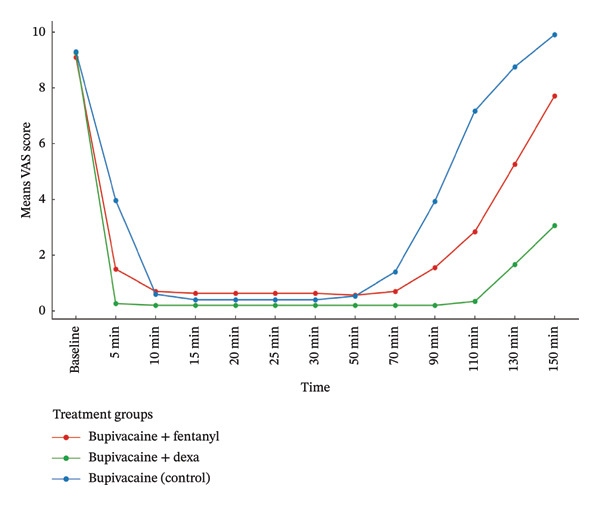
Mean VAS scores over time by treatment group.

Pain intensity, assessed by the VAS, was consistently and significantly lower in the dexmedetomidine group throughout the study period. As shown in Figure 2, the mean VAS scores over time differed significantly among the study groups. Group BD (dexmedetomidine) consistently demonstrated lower pain scores compared to Group BF (fentanyl) and Group B (control), reflecting a more effective and sustained analgesic effect. These visual trends align with the results of the repeated‐measures ANOVA, which revealed a significant main effect of group (*p* < 0.001, partial *η* ^2^ = 0.69), indicating a substantial difference in overall pain levels between groups. Furthermore, a significant time × group interaction (*p* < 0.001, partial *η* ^2^ = 0.67) was observed, confirming that the pattern of pain reduction over time varied across treatments. Group BD showed the most rapid and sustained decrease in pain intensity, while Group B exhibited the slowest decline. These differences were further supported by post hoc pairwise comparisons at multiple time points (Table 3).

**TABLE 3:** Repeated‐measures ANOVA results for VAS scores (5–150 min).

Effect	*p* value	Partial *η* ^2^	Bonferroni post hoc comparisons (*p* value)	Interpretation
Time	**< 0.001**	0.72 (large)	Pain scores significantly decreased from 5–150 min (all pairs *p* < 0.01)	Significant pain reduction over time across all groups

Group	**< 0.001**	0.69 (large)	BD vs control: *p* < 0.001 Fentanyl vs control: *p* = 0.004 BD vs fentanyl: *p* = 0.03	Significant differences in average pain levels between groups, with BD having the best pain control

Time × Group	**< 0.001**	0.67 (large)	Interaction post hoc: At 15 min: BD vs control: *p* = 0.002 Fentanyl vs control: *p* = 0.05 BD vs fentanyl: *p* = 0.04 At 60 min: BD vs control: *p* < 0.001 Fentanyl vs control: *p* = 0.01 BD vs fentanyl *p* = 0.02 At 150 min: BD vs control: *p* = 0.005 Fentanyl vs control: *p* = 0.12 (NS) BD vs fentanyl: *p* = 0.09 (NS)	Pain trajectories differ: BD group shows faster and more sustained pain reduction; fentanyl intermediate; control slowest

*Note:* Model structure for repeated‐measures ANOVA results for VAS pain scores—within‐subjects factor: time (12 postintervention time points)–between‐subjects factor: Group (control vs. Dexmedetomidine vs. fentanyl)‐dependent variable: VAS pain score (0–10). Partial *η* ^2^: partial eta squared > 0.14 indicates large effect sizes. Bold represents significant *p* value.

As shown in Table 4, repeated‐measures ANOVA revealed significant main effects of time for all measured hemodynamic parameters—pulse rate, mean arterial pressure (MAP), RR, and SpO_2_—indicating notable within‐subject changes over the observation period (all *p* < 0.05, partial *η* ^2^ ranging from 0.60 to 0.75, denoting large effect sizes).

**TABLE 4:** Repeated‐measures ANOVA with Bonferroni post hoc comparisons for intraoperative hemodynamic parameters (5–150 min).

Parameter	Effect	*p* value	Partial *η* ^2^	Bonferroni post hoc comparisons (*p* value)	Interpretation
Pulse rate	Time	**< 0.001**	0.75 (large)	—Significant—	PR changed significantly over time
	Group	**< 0.001**	0.45 (large)	BD vs control: < 0.001 Fentanyl vs control: 0.245 BD vs fentanyl: < 0.001	BD group had significantly lower PR than others
	Time × Group	**< 0.001**	0.30 (large)	—Significant—	Change pattern over time differed by group

MAP	Time	**< 0.001**	0.65 (large)	—Significant—	MAP declined, then recovered over time
	Group	**< 0.001**	0.07 (medium)	BD vs control: 0.019 Fentanyl vs control: 0.538 BD vs fentanyl: 0.021	Small but significant differences between BD and others
	Time × Group	0.650	0.01 (small)	—NS—	Groups followed similar MAP patterns

RR	Time	**< 0.001**	0.60 (large)	—Significant—	RR declined initially, then recovered
	Group	**< 0.001**	0.15 (large)	BD vs control: < 0.001 Fentanyl vs control: 0.132 BD vs fentanyl: 0.028	BD had a slightly lower RR than the others
	Time × Group	0.083	0.04 (small)	—NS—	Group patterns over time were not significantly different

SpO_2_	Time	**0.017**	0.10 (medium)	—Significant—	Small changes over time
	Group	**0.002**	0.08 (medium)	BD vs control: 0.014 Fentanyl vs control: 0.003 BD vs fentanyl: 0.425	BD and fentanyl groups had slightly lower SpO_2_ than the control
	Time × Group	**0.002**	0.08 (medium)	—Significant—	Patterns of SpO_2_ changes differed by group

*Note:* Repeated‐measures ANOVA with Bonferroni post hoc comparisons for intraoperative hemodynamic parameters (5–150 min): measure the same subjects multiple times (e.g., at 5, 10, …, 150 min) and want to compare trends within groups over time and between groups across time; differences are group‐wise post hoc *p* values; partial *η* ^2^ values are estimated. Bold represents significant *p* value.

A significant group effect was also observed for all parameters, especially pulse rate and RR, with Group BD (dexmedetomidine) consistently demonstrating lower mean values compared to the other groups (pulse rate: *p* < 0.001, partial *η* ^2^ = 0.45; RR: *p* < 0.001, partial *η* ^2^ = 0.15). Post hoc comparisons confirmed that these differences were primarily between Group BD and the other two groups, suggesting a sympatholytic effect of dexmedetomidine.

Importantly, the time × group interaction was statistically significant for pulse rate and SpO_2_ (*p* < 0.001 and *p* = 0.002, respectively), indicating that the trajectory of these variables over time differed among groups. However, the interaction effect was nonsignificant for MAP (*p* = 0.650) and RR (*p* = 0.083), suggesting that while initial values varied, the trends over time were relatively parallel across the groups.

Figure 3 graphically supported these findings, demonstrating that Group BD maintained a slightly lower but stable pulse rate and RR throughout the observation period, without causing clinically significant bradycardia or desaturation. These trends align with the pharmacodynamic profile of dexmedetomidine and confirm that the hemodynamic changes observed were statistically significant but clinically well‐tolerated.

FIGURE 3Mean maternal hemodynamic parameters over time by treatment groups.

**Figure:**
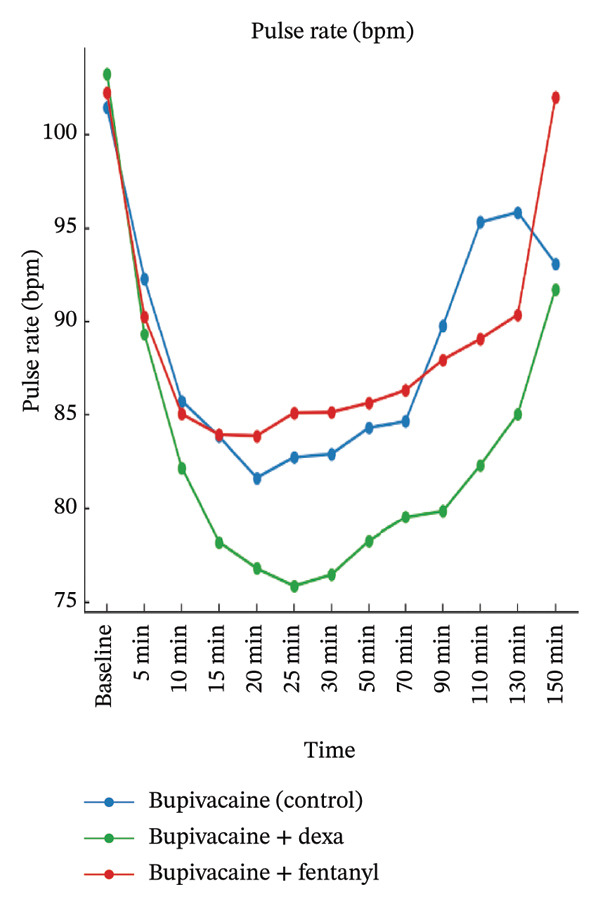
(a)

**Figure:**
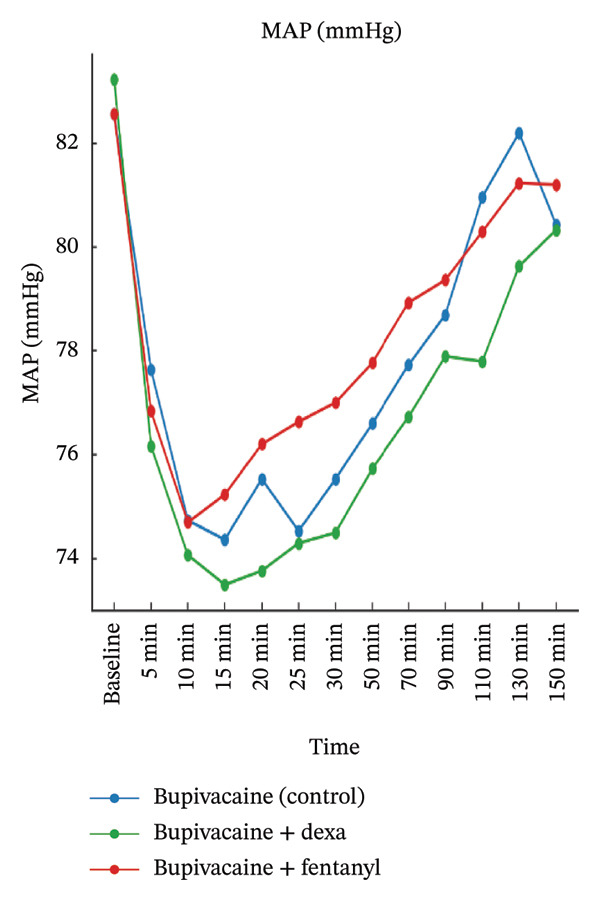
(b)

Adverse effects varied by group. Group BF, which received intrathecal fentanyl, had a higher incidence of pruritus (30%) and nausea (20%), consistent with known opioid‐related side effects. In contrast, Group BD showed a lower incidence of side effects, limited primarily to manageable bradycardia. The control group (Group B) had minimal adverse events, likely due to the absence of adjuvant medications. No vomiting, respiratory depression, or serious complications were reported in any group. No episodes of pathological fetal bradycardia were recorded in any group during the monitoring period.

Neonatal outcomes, assessed by Apgar scores at 1 and 5 min, were comparable across all three groups and within normal clinical limits. There were no statistically significant differences (*p* > 0.05), indicating that none of the tested analgesic regimens adversely affected neonatal well‐being.

## 4. Discussion

This randomized controlled trial compared the analgesic efficacy, maternal satisfaction, and safety profile of intrathecal bupivacaine alone, bupivacaine with fentanyl, and bupivacaine with dexmedetomidine for labor analgesia. The findings clearly demonstrate that adding dexmedetomidine to low‐dose intrathecal bupivacaine provided the most favorable balance between rapid onset, prolonged duration of analgesia, high maternal satisfaction, and acceptable hemodynamic stability. These results strengthen the evidence supporting dexmedetomidine as a promising nonopioid adjuvant in obstetric anesthesia.

The superior performance of dexmedetomidine in this study is consistent with previous clinical observations in both obstetric and nonobstetric populations. Low doses of intrathecal dexmedetomidine (3–5 μg) have been shown to effectively prolong sensory block duration and enhance the quality of analgesia without causing excessive sedation or motor blockade [13–15]. The enhanced analgesic effect is primarily attributed to its highly selective *α*_2_‐adrenergic agonist mechanism, which modulates pain at multiple levels of the neuraxis. By inhibiting presynaptic norepinephrine release and postsynaptic hyperpolarization of dorsal horn neurons, dexmedetomidine reduces nociceptive transmission through both spinal and supraspinal mechanisms [9, 16]. This dual action results in a synergistic effect with local anesthetics, producing dense and long‐lasting sensory blockade with minimal motor involvement, an ideal profile for labor analgesia.

Compared with fentanyl, dexmedetomidine offered a distinct pharmacological advantage. Although fentanyl is widely recognized for its potent and rapid analgesic effect when added to local anesthetics, it remains associated with opioid‐related side effects such as nausea, vomiting, pruritus, and, in rare cases, respiratory depression [8]. In contrast, dexmedetomidine achieved comparable, and in many cases superior, analgesia without these drawbacks. In the present study, parturients receiving dexmedetomidine reported greater comfort and satisfaction, likely reflecting both effective pain control and minimal adverse effects. These findings correspond with the work of Bajwa et al. [14], who observed improved postoperative comfort and reduced need for rescue analgesics when dexmedetomidine was used as an intrathecal adjuvant.

An important clinical consideration is whether the duration of spinal analgesia was sufficient to cover the entire labor process. In our cohort, the analgesic duration in Group BD (124.2 ± 15.4 min) and Group BF (106.2 ± 18.9 min) exceeded the mean total labor duration (78–86 min across groups), indicating that most parturients in these groups delivered while still under effective analgesia. Although time to full cervical dilation was not a prespecified endpoint, the nonsignificant difference in total labor duration across groups (*p* = 0.21) indicates that none of the analgesic regimens significantly influenced the rate of labor progression, consistent with existing evidence that single‐shot neuraxial analgesia does not delay the active phase of labor when administered at appropriate cervical dilation. Furthermore, no significant correlation was observed between analgesic duration and time to delivery (Spearman *ρ* = 0.11, *p* = 0.29), confirming that the mode of analgesia did not confound obstetric outcomes. In future studies, we recommend prospectively recording time to full cervical dilation as a discrete endpoint to better characterize the interaction between analgesic duration and labor progression.

Another noteworthy observation in this study was the hemodynamic profile associated with dexmedetomidine. While mild reductions in HR and RR were statistically significant, they remained clinically acceptable and easily managed with standard interventions. This effect aligns with the central sympatholytic action of *α*_2_‐agonists, which decrease sympathetic outflow and reduce circulating catecholamines. Previous research by Al‐Mustafa et al. [15] and Kanazi et al. [13] similarly documented mild bradycardia following intrathecal dexmedetomidine, but without serious sequelae. Importantly, MAP remained stable, and no episodes of clinically significant hypotension or desaturation were encountered, confirming the cardiovascular safety of low‐dose dexmedetomidine in laboring women.

From a clinical standpoint, these findings have significant implications, particularly for obstetric units in resource‐limited settings. SSSA is a simple, reliable, and cost‐effective technique that can provide excellent pain relief during labor when continuous epidural services are unavailable. In such contexts, dexmedetomidine represents an attractive alternative to opioids because it provides prolonged analgesia with fewer side effects and does not require specialized monitoring for respiratory depression. Moreover, the ability to maintain maternal consciousness and mobility during labor promotes active participation in the birthing process and contributes to the high satisfaction scores observed.

The safety of intrathecal dexmedetomidine for the fetus was reaffirmed in this study, as neonatal Apgar scores remained within normal limits and were comparable across groups. The minimal placental transfer of dexmedetomidine, combined with the low dose used, likely explains the absence of neonatal adverse effects, consistent with prior evidence [9, 14]. This supports its suitability for obstetric use under careful monitoring.

### 4.1. Limitations

Despite these encouraging findings, several limitations should be acknowledged. The study was conducted at a single center, which may limit the generalizability of results. The single‐shot spinal technique, while practical, does not allow dose titration or extension of analgesia for prolonged labor. Cervical effacement was not systematically recorded in all participants, preventing its inclusion in the comparative table; this is acknowledged as a limitation and will be incorporated as a mandatory variable in future protocols. Additionally, discharge time was not prospectively recorded in our dataset and thus cannot be reported; we note this as a limitation and recommend including discharge timing in future protocols. Additionally, subjective factors such as maternal anxiety and previous labor experience, which could influence satisfaction scores, were not formally assessed. Intrathecal dexmedetomidine is an off‐label use not approved by the FDA and is approached cautiously by major journals due to potential neurotoxicity concerns. However, this study was ethically approved, registered, and conducted in accordance with international standards. Existing evidence, including meta‐analyses, supports its efficacy and safety at low doses (3–5 μg), with no demonstrated neurotoxicity in preclinical studies. Nevertheless, its use should remain restricted to settings with strict ethical oversight and close monitoring until further long‐term safety data and regulatory approval are established. Future multicenter studies should address these limitations and explore continuous or repeated intrathecal administration strategies. Further research could also examine the combination of dexmedetomidine with other adjuvants or reduced local anesthetic doses to optimize efficacy while minimizing potential bradycardia.

## 5. Conclusion

The addition of 5 μg dexmedetomidine to intrathecal bupivacaine significantly improved the quality and duration of labor analgesia, provided stable hemodynamics, and enhanced maternal satisfaction without compromising neonatal outcomes. These results confirm that dexmedetomidine is a safe and effective nonopioid alternative to fentanyl for single‐shot spinal labor analgesia. Its use may be particularly valuable in resource‐limited environments where simplicity, safety, and prolonged analgesia are essential for improving maternal birthing experiences.

## Author Contributions

Ayman Mohamady Eldemrdash: conceptualization, methodology, supervision, project administration, and writing–review and editing.

Taha Tairy Dardeer Elsawy: data curation, investigation, formal analysis, and writing–original draft.

Zaher Zaki Zaher: investigation, resources, and writing–review and editing.

Jehan Sadek Younan: data collection, patient assessment, and writing–original draft.

Ahmed Mohammed Reda Ragheb: statistical analysis, visualization, and writing–review and editing.

## Funding

There was no external funding in the preparation of this manuscript.

## Consent

The authors have nothing to report.

## Conflicts of Interest

The authors declare no conflicts of interest.

## Data Availability

The datasets generated and analyzed during the current study are not publicly available due to institutional restrictions, but are available from the corresponding author on reasonable request.
